# Supplementary material for: Polyhedral Distortions and Unusual Magnetic Order in Spinel FeMn2O4
Source: Chem Mater. 2023 Mar 14;35(6):2330–41. doi: 10.1021/acs.chemmater.2c03182 (PMC11008780; doi:10.1021/acs.chemmater.2c03182)
Supplement: Supplementary file 1 — cm2c03182_si_001.pdf [file cm2c03182_si_001.pdf]

Supporting Information for

Polyhedral distortions and unusual magnetic order in spinel $FeMn_2O_4$

Qiang Zhang,[*,†,‡] Wei Tian,[‡] Roshan K. Nepal,[†] Ashfia Huq,[‡,§] Stephen Nagler,[‡] J. F. DiTusa,[†] and Rongying Jin[*,†,¶]

†*Department of Physics and Astronomy, Louisiana State University, Baton Rouge, Louisiana 70803, USA*

‡*Neutron Scattering Division, Oak Ridge National Laboratory, Oak Ridge, Tennessee 37831, USA*

¶*Center for Experimental Nanoscale Physics, Department of Physics and Astronomy, University of South Carolina, Columbia, SC 29208, USA*

§*Current address: Sandia National Laboratories, Livermore, CA 94551, USA*

E-mail: zhangq6@ornl.gov; rjin@mailbox.sc.edu

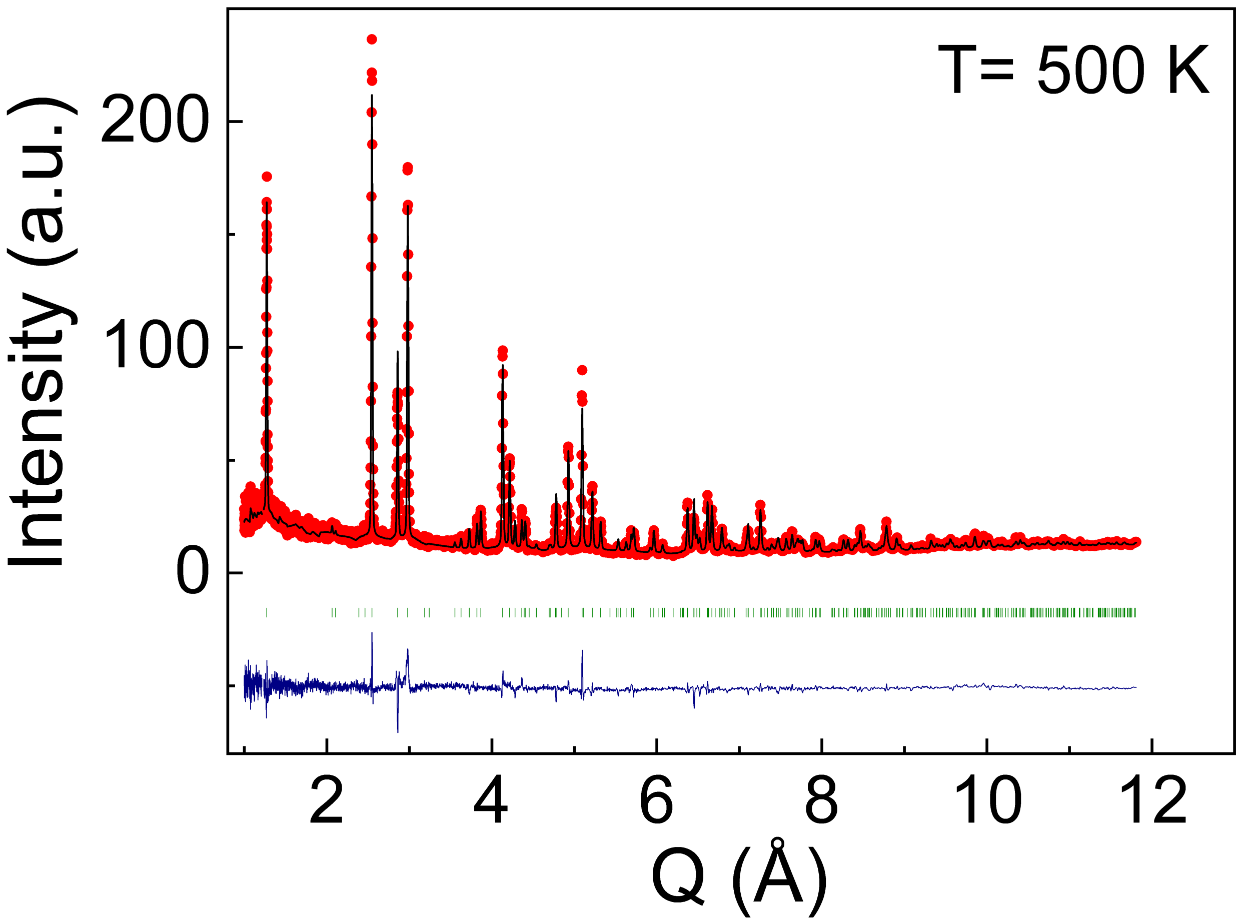

Figure S1: Rietveld refinement fits to high resolution neutron diffraction patterns at 500 K after the thermal cycle.

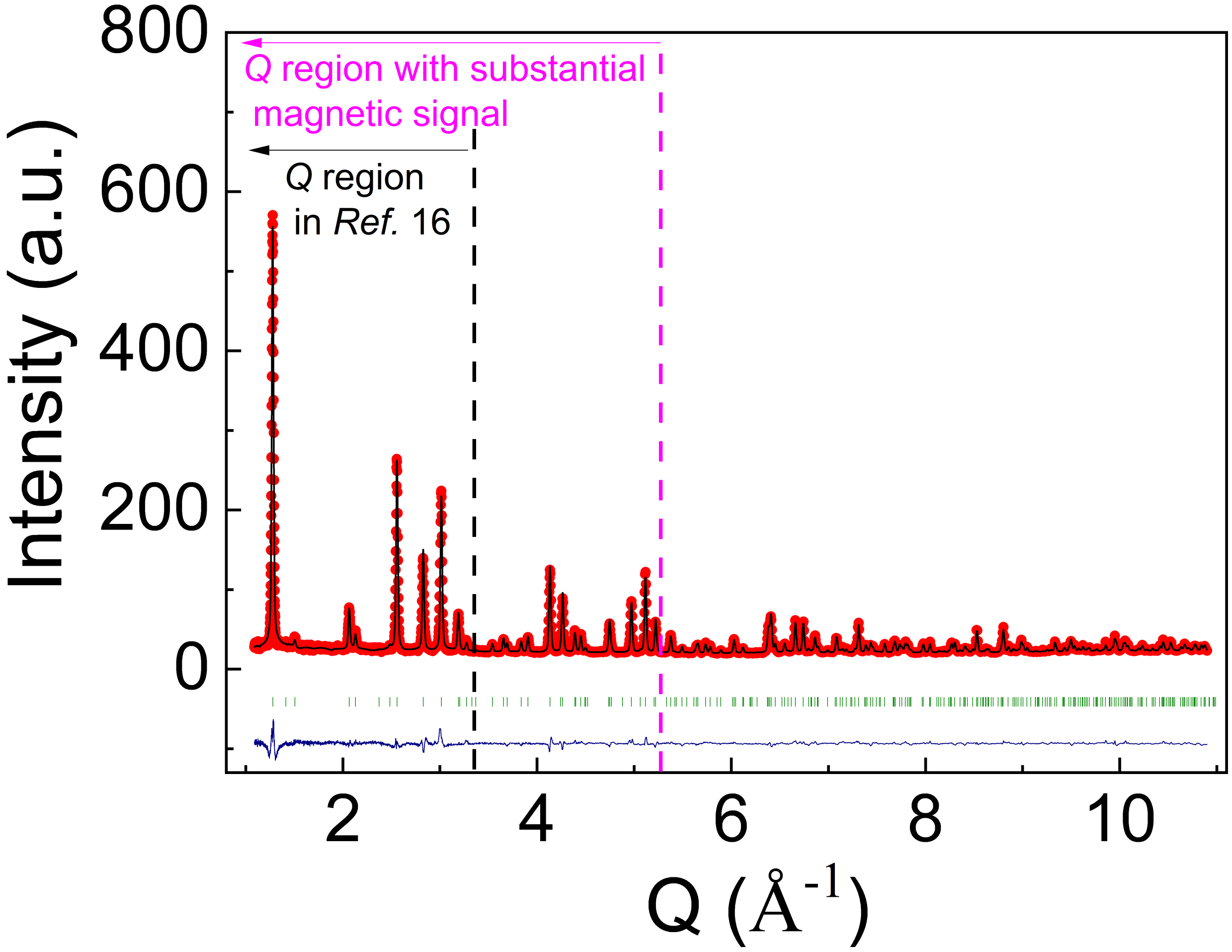

Figure S2: Comparison of Q regions between the neutron data in Ref. 13 of the main text and our POWGEN data at 10 K.
